# Supplementary material for: Structure and inhibition of the human lysosomal transporter Sialin
Source: Nat Commun. 2024 May 23;15:4386. doi: 10.1038/s41467-024-48535-3 (PMC11116495; doi:10.1038/s41467-024-48535-3)
Supplement: Supplementary file 1 — Supplementary Information [file 41467_2024_48535_MOESM1_ESM.pdf]

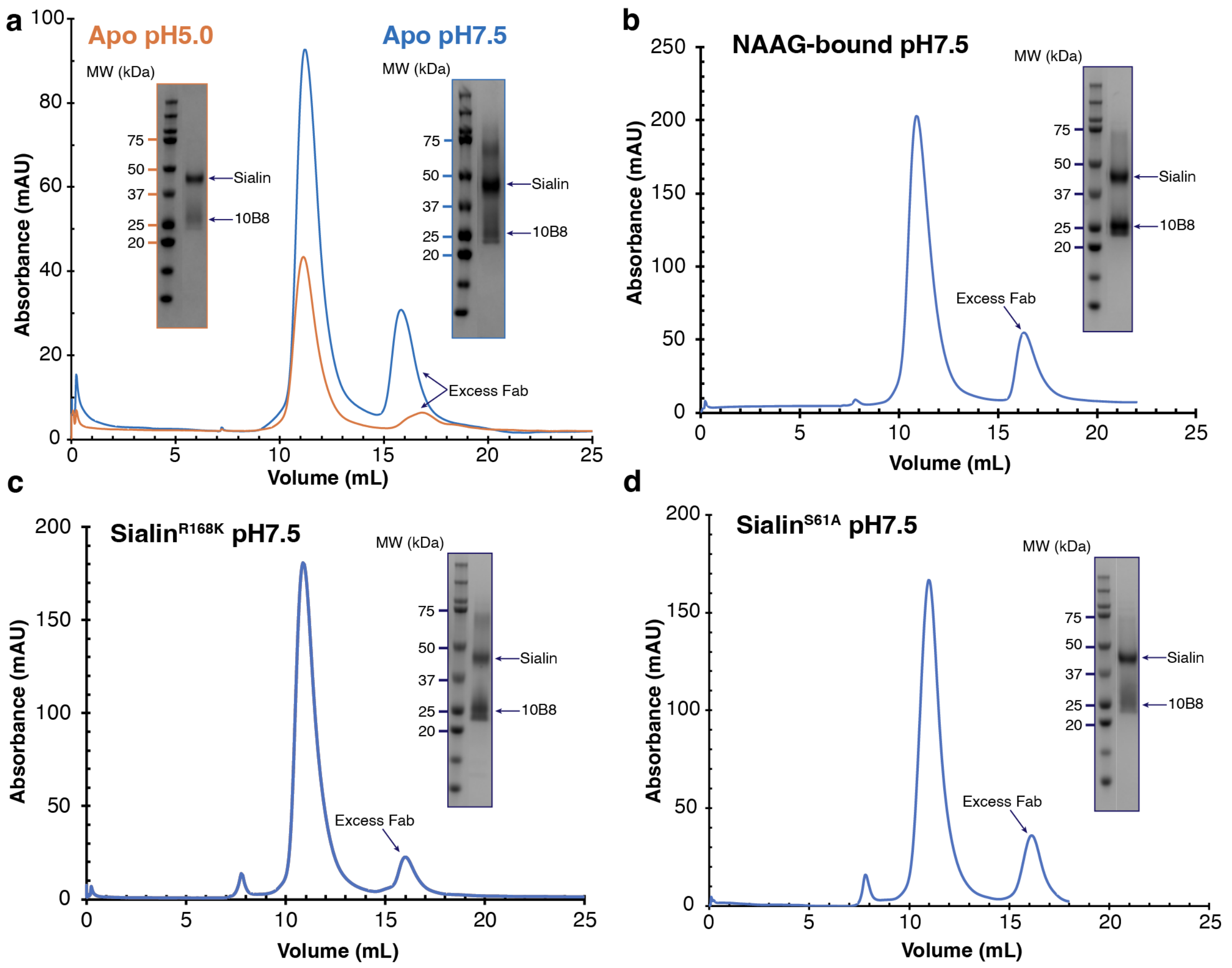

Supplementary Fig. 1: The purification of the human Sialin constructs used for structural studies.

a Representative Superdex 200 Increase 10/300 GL gel-filtration chromatogram of Fab^{10B8} bound Sialin at pH 5.0 (orange) and representative Superdex 200 Increase 10/300 GL gel-filtration chromatogram of Fab^{10B8} bound Sialin at pH 7.5 (blue). **b** Representative Superdex 200 Increase 10/300 GL gel-filtration chromatogram of Fab^{10B8} bound Sialin in the presence of 0.2 mM NAAG at pH 7.5. **c** Representative Superdex 200 Increase 10/300 GL gel-filtration chromatogram of Sialin^{R168K} at pH 7.5. **d** Representative Superdex 200 Increase 10/300 GL gel-filtration chromatogram of Sialin^{S61A} at pH 7.5. The peak fractions from each purification are shown on SDS-PAGE.

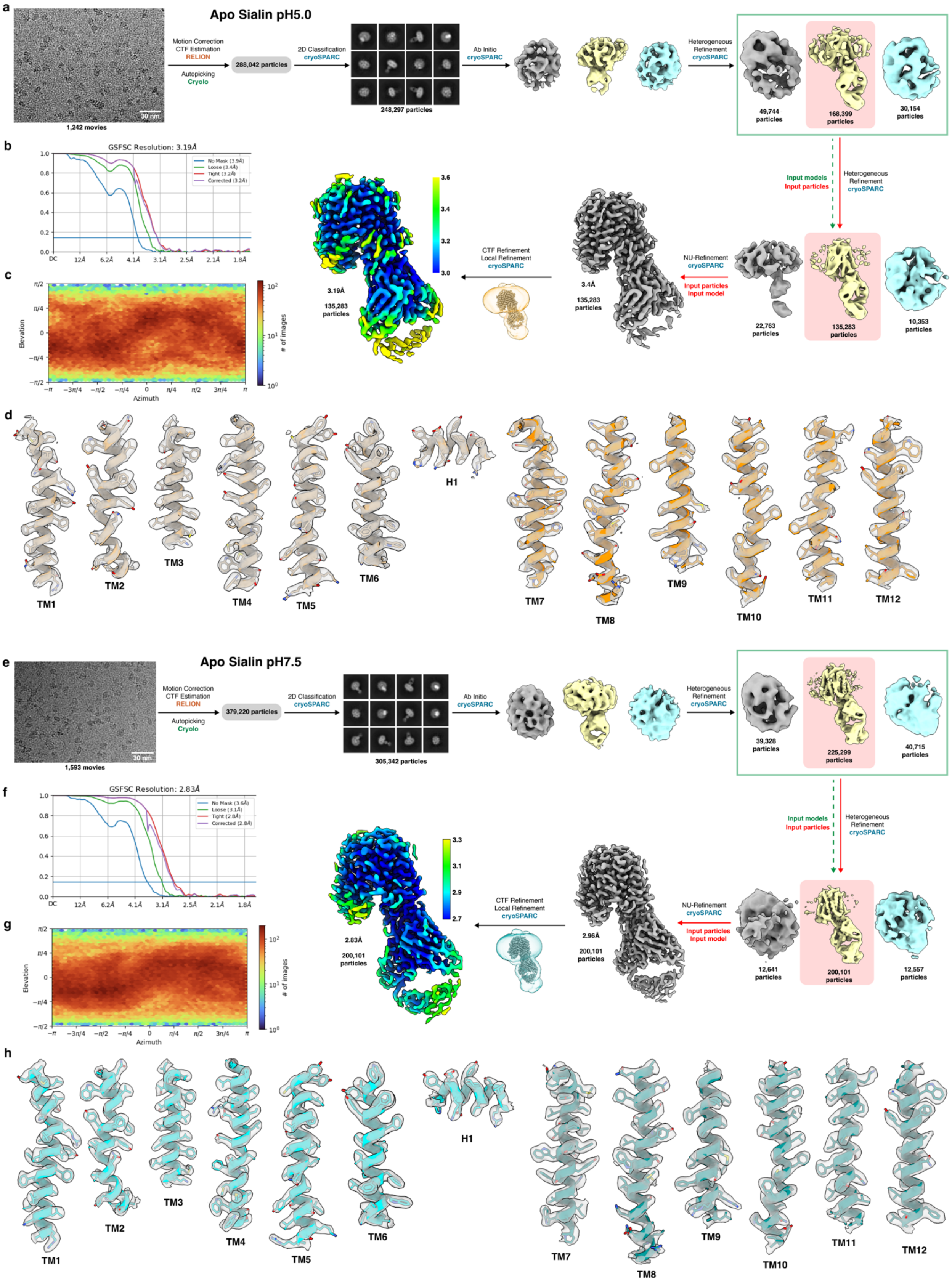

a
Apo Sialin pH5.0
30 nm
1,242 movies
Motion Correction
CTF Estimation
RELION
Autopicking
Cryolo
288,042 particles
2D Classification
cryoSPARC
248,297 particles
Ab Initio
cryoSPARC
Heterogeneous
Refinement
cryoSPARC
49,744
particles
168,399
particles
30,154
particles
Input models
Input particles
Heterogeneous
Refinement
cryoSPARC
b
GSFSC Resolution: 3.19Å
No Mask (3.9Å)
Loose (3.4Å)
Tight (3.2Å)
Corrected (3.2Å)
c
Elevation
Azimuth
# of images
3.19Å
135,283
particles
CTF Refinement
Local Refinement
cryoSPARC
3.4Å
135,283
particles
NU-Refinement
cryoSPARC
Input particles
Input model
22,763
particles
135,283
particles
10,353
particles
d
TM1
TM2
TM3
TM4
TM5
TM6
H1
TM7
TM8
TM9
TM10
TM11
TM12
e
Apo Sialin pH7.5
30 nm
1,593 movies
Motion Correction
CTF Estimation
RELION
Autopicking
Cryolo
379,220 particles
2D Classification
cryoSPARC
305,342 particles
Ab Initio
cryoSPARC
Heterogeneous
Refinement
cryoSPARC
39,328
particles
225,299
particles
40,715
particles
Input models
Input particles
Heterogeneous
Refinement
cryoSPARC
f
GSFSC Resolution: 2.83Å
No Mask (3.6Å)
Loose (3.1Å)
Tight (2.8Å)
Corrected (2.8Å)
g
Elevation
Azimuth
# of images
2.83Å
200,101
particles
CTF Refinement
Local Refinement
cryoSPARC
2.96Å
200,101
particles
NU-Refinement
cryoSPARC
Input particles
Input model
12,641
particles
200,101
particles
12,557
particles
h
TM1
TM2
TM3
TM4
TM5
TM6
H1
TM7
TM8
TM9
TM10
TM11
TM12

Supplementary Fig. 2: Cryo-EM workflow and analysis of Sialin in the lumen-open apo conformation.

a Summary of the image processing procedures of apo Sialin purified at pH 5.0. The final map is colored by local resolution estimation using cryoSPARC. The micrograph is a cropped representative image of the protein sample on the grid. **b** Fourier shell correlation (FSC) curves of the final reconstruction from cryoSPARC. **c** Representation of the angular distribution of the particles used in the final reconstruction from cryoSPARC. **d** Major structural features of apo Sialin at pH 5.0 with structural model shown as cartoons and colored as in Fig. 1, and map density in gray. **e** Summary of the image processing procedures of apo Sialin purified at pH 7.5. The final map is colored by local resolution estimation using cryoSPARC. The micrograph is a cropped representative image of the protein sample on the grid. **f** Fourier shell correlation (FSC) curves of the final reconstruction from cryoSPARC. **g** Representation of the angular distribution of the particles used in the final reconstruction from cryoSPARC. **h** Major structural features of apo Sialin at pH 7.5 with structural model shown as cartoons and colored as in Fig 1, and map density in gray.

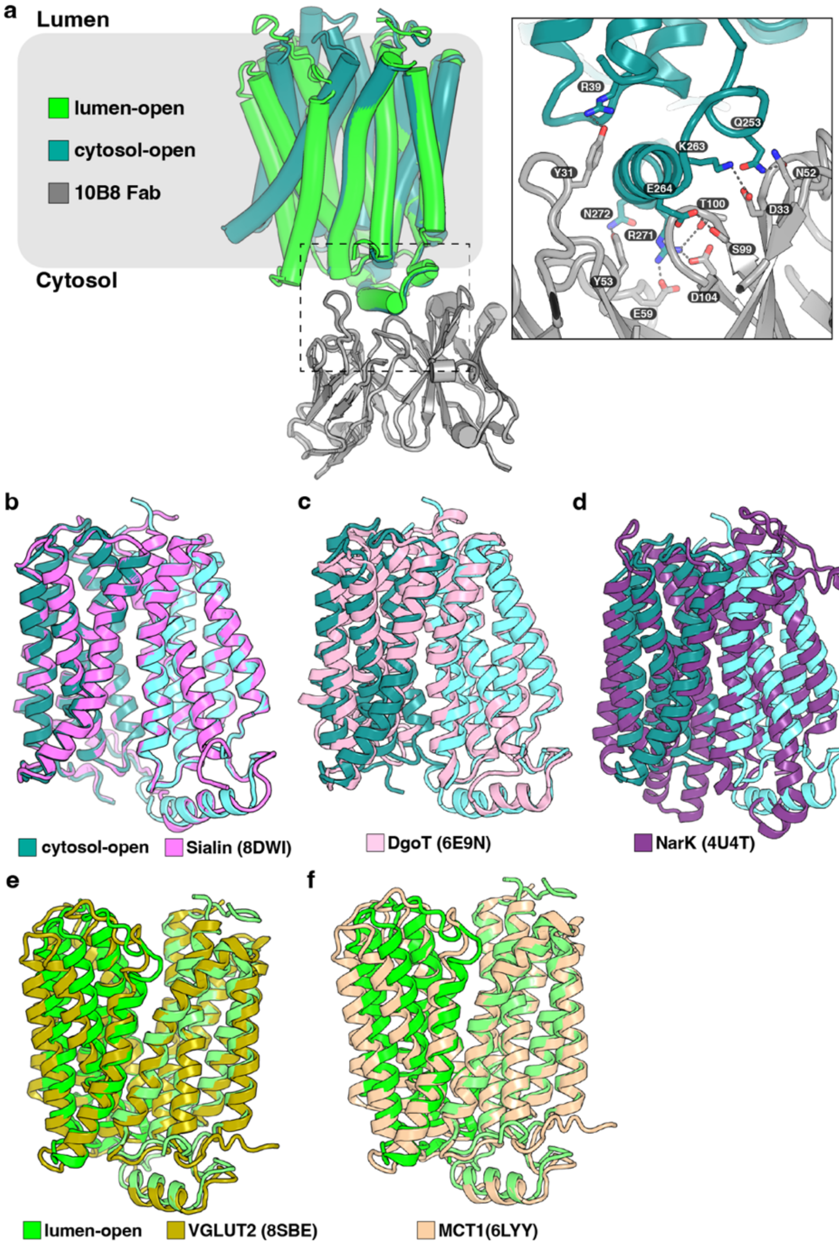

Supplementary Fig. 3: Sialin-Fab^{10B8} details and structural comparisons with other MFS transporters.

a The interaction details between Fab^{10B8} and Sialin. The structural comparison between lumen-open and cytosol-open states shows that Fab^{10B8} does not affect the conformational change of Sialin. **b** Structural comparison of Sialin^{WT} and the recently determined apo-structure of Sialin. **c** Structural comparison of Sialin^{WT} and DogT. **d** Structural comparison of Sialin^{WT} and NarK. **e** Structural comparison of Sialin^{R168K} and VGLUT2. **f** Structural comparison of Sialin^{R168K} and MCT1.

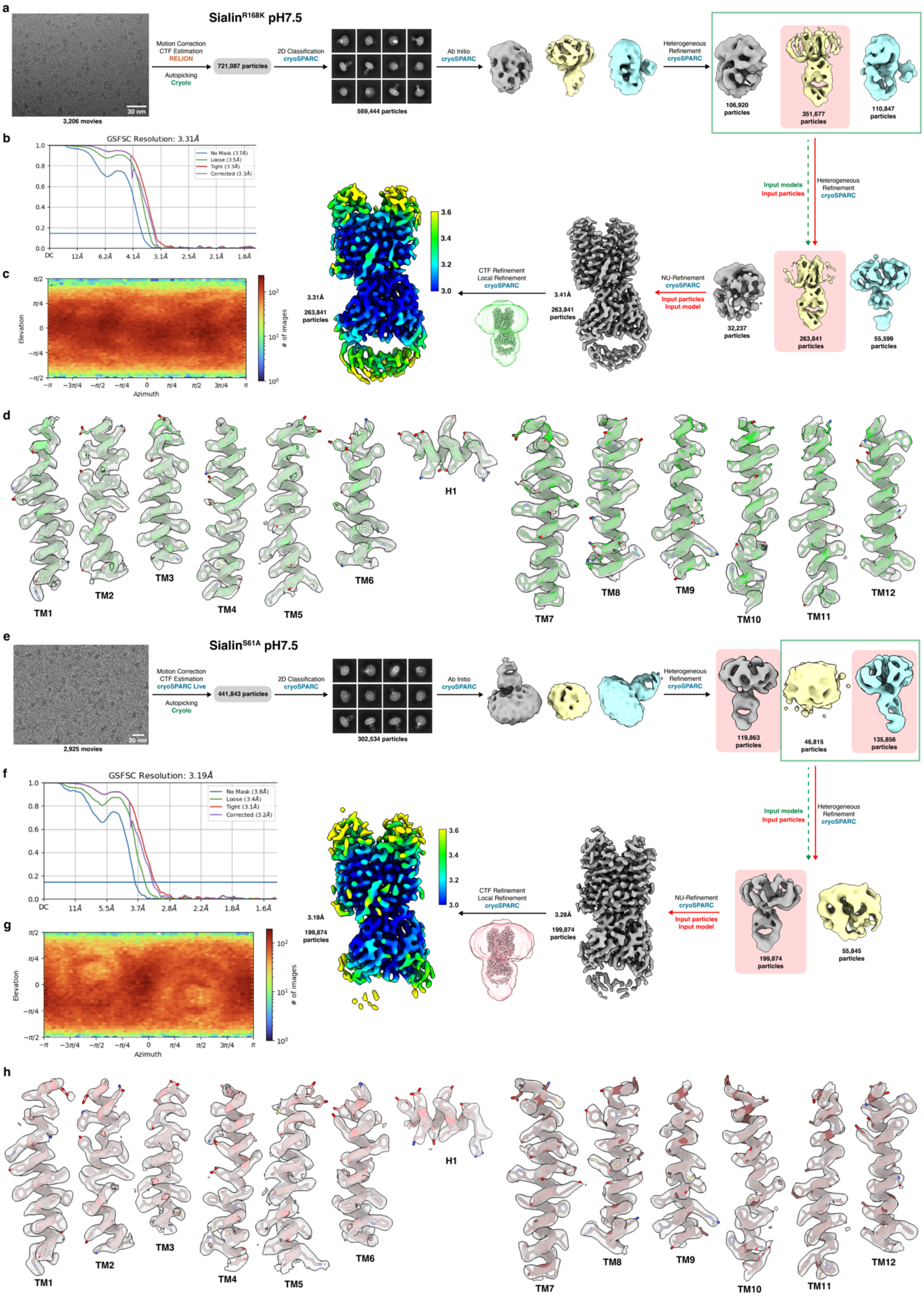

a
Sialin R168K pH7.5
30 nm
3,206 movies
Motion Correction
CTF Estimation
RELION
Autopicking
Cryolo
721,087 particles
2D Classification
cryoSPARC
569,444 particles
Ab Initio
cryoSPARC
Heterogeneous Refinement
cryoSPARC
106,920 particles
351,677 particles
110,847 particles
Input models
Input particles
Heterogeneous Refinement
cryoSPARC
32,237 particles
263,841 particles
55,599 particles
NU-Refinement
cryoSPARC
Input particles
Input model
3.41Å
263,841 particles
CTF Refinement
Local Refinement
cryoSPARC
3.31Å
263,841 particles
3.6
3.4
3.2
3.0
b
GSFSC Resolution: 3.31Å
No Mask (3.7Å)
Loose (3.5Å)
Tight (3.3Å)
Corrected (3.3Å)
c
Elevation
Azimuth
# of images
d
TM1
TM2
TM3
TM4
TM5
TM6
H1
TM7
TM8
TM9
TM10
TM11
TM12
e
Sialin S61A pH7.5
30 nm
2,925 movies
Motion Correction
CTF Estimation
cryoSPARC Live
Autopicking
Cryolo
441,843 particles
2D Classification
cryoSPARC
302,534 particles
Ab Initio
cryoSPARC
Heterogeneous Refinement
cryoSPARC
119,863 particles
46,815 particles
135,856 particles
Input models
Input particles
Heterogeneous Refinement
cryoSPARC
199,874 particles
55,845 particles
NU-Refinement
cryoSPARC
Input particles
Input model
3.28Å
199,874 particles
CTF Refinement
Local Refinement
cryoSPARC
3.19Å
199,874 particles
f
GSFSC Resolution: 3.19Å
No Mask (3.8Å)
Loose (3.4Å)
Tight (3.1Å)
Corrected (3.2Å)
g
Elevation
Azimuth
# of images
h
TM1
TM2
TM3
TM4
TM5
TM6
H1
TM7
TM8
TM9
TM10
TM11
TM12

Supplementary Fig. 4: Cryo-EM workflow and analysis of Sialin^{R168K} and Sialin^{S61A} in the lumen-open apo conformation.

a Summary of the image processing procedures of apo Sialin^{R168K} purified at pH 7.5. The final map is colored by local resolution estimation using cryoSPARC. The micrograph is a cropped representative image of the protein sample on the grid. **b** Fourier shell correlation (FSC) curves of the final reconstruction from cryoSPARC. **c** Representation of the angular distribution of the particles used in the final reconstruction from cryoSPARC. **d** Major structural features of apo Sialin^{R168K} at pH 7.5 with structural model shown as cartoons and colored as in Fig 1, and map density in gray. **e** Summary of the image processing procedures of apo Sialin^{S61A} purified at pH 7.5. The final map is colored by local resolution estimation using cryoSPARC. The micrograph is a cropped representative image of the protein sample on the grid. **f** Fourier shell correlation (FSC) curves of the final reconstruction from cryoSPARC. **g** Representation of the angular distribution of the particles used in the final reconstruction from cryoSPARC. **h** Major structural features of apo Sialin^{S61A} at pH 7.5 with structural model shown as cartoons and colored as in Fig 1, and map density in gray.

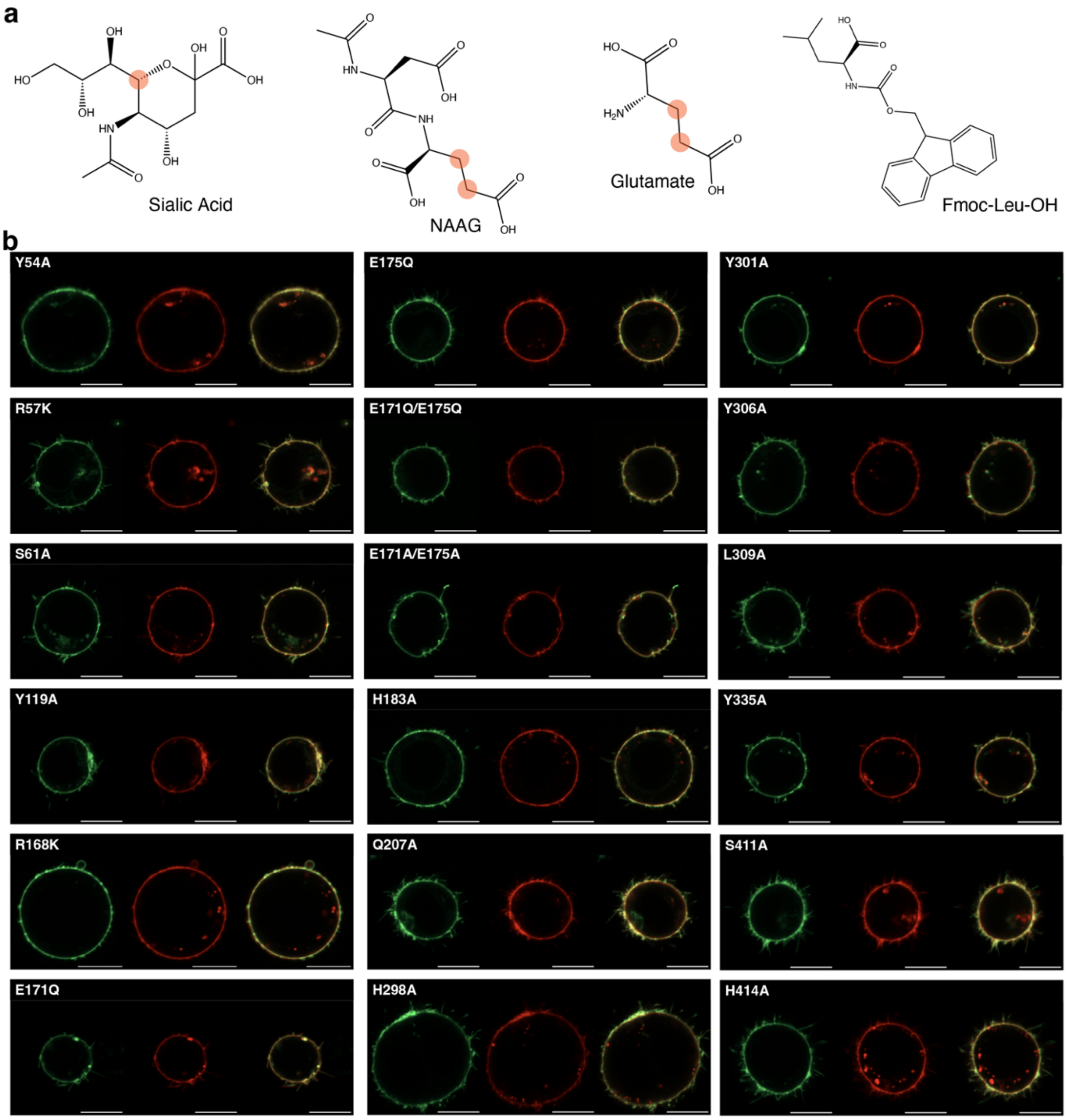

Supplementary Fig. 5: Representative images of the cellular localization of Sialin$^{\text{GPF/LL}}$ mutants.

a Chemical structures of molecules used in cell-based transport assays (from left to right): N-acetylneuraminic acid (NANA), N-acetylaspartylglutamate (NAAG), Glutamate, and Fmoc-Leu-OH. The carbon atoms with tritiated hydrogens are indicated by orange circles on the respective molecules. **b** HEK293 cells co-expressing the indicated Sialin$^{\text{GFP/LL}}$ mutants (in green), and Spns2-mCherry (in red) as a positive control, and merged images (in yellow). Scale bar shown on each image is 10 μm. Expression experiments were repeated a total of 3 times.

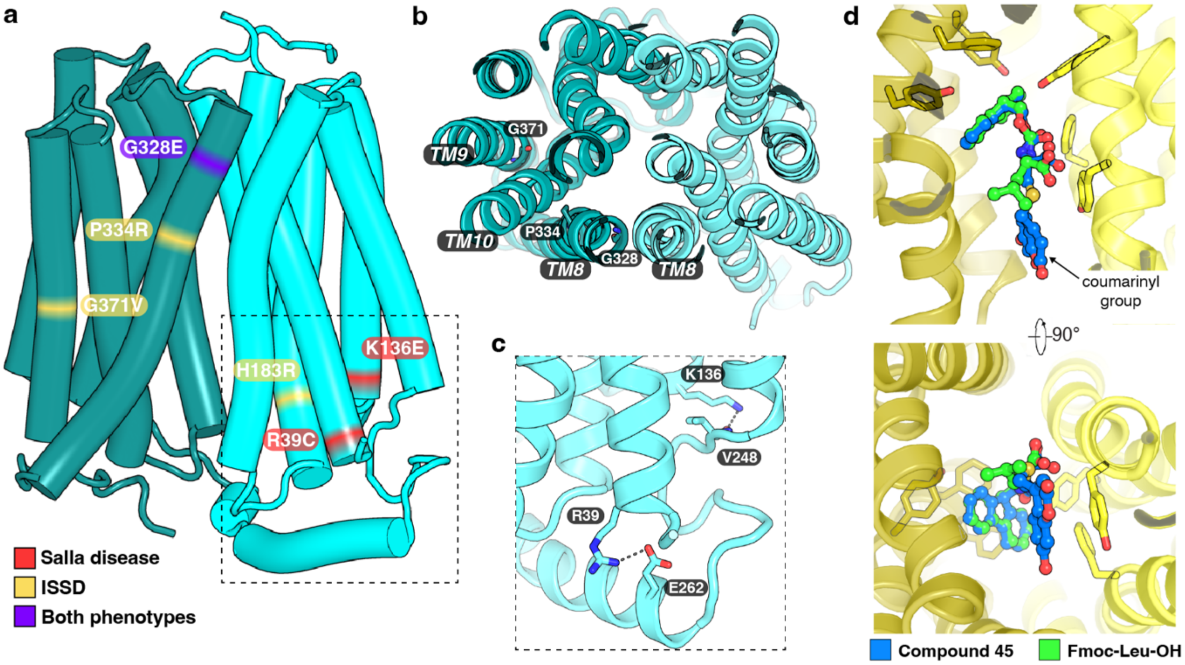

Supplementary Fig. 6: The putative mechanism of disease-causing Sialin mutations.

a Disease-causing mutations classified and mapped onto the cytosol-open model of Sialin. **b** and **c** The putative mechanism of ISSD and Salla disease mutations. TMs and related residues are labeled. The hydrophilic interactions are indicated by dashed lines. **d** Compound 45 modeled into the Fmoc-Leu-OH bound Sialin structure. Compound 45 is shown as sticks in blue.

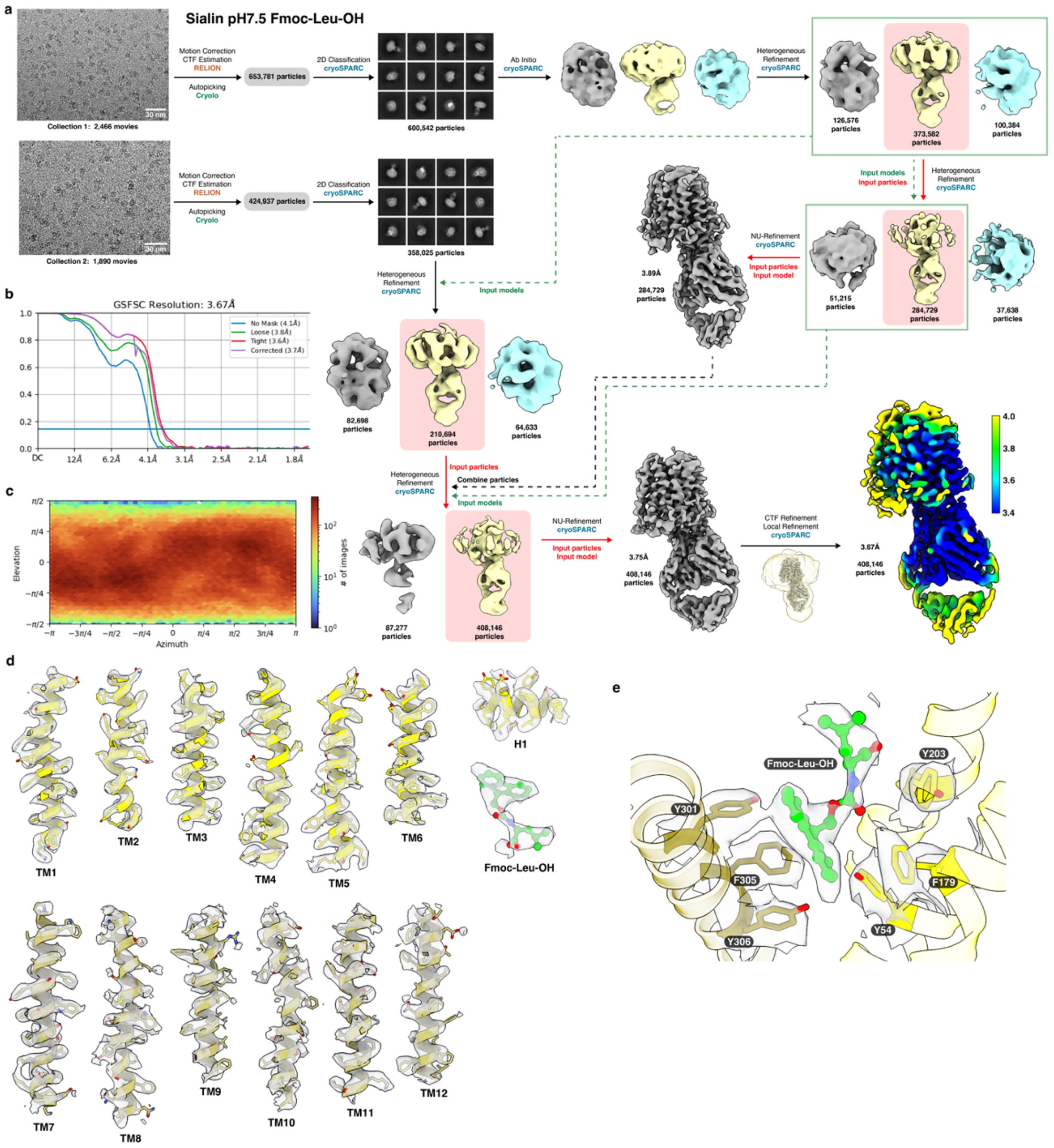

Supplementary Fig. 7: Cryo-EM workflow and analysis of Fmoc-Leu-OH-bound Sialin.

a Summary of the image processing procedures of Fmoc-Leu-OH-bound Sialin purified at pH 7.5. The final map is colored by local resolution estimation using cryoSPARC. The micrographs are cropped representative images of the protein sample on the grids. **b** Fourier shell correlation (FSC) curves of the final reconstruction from cryoSPARC. **c** Representation of the angular distribution of the particles used in the final reconstruction from cryoSPARC. **d** Major structural features of Fmoc-Leu-OH-bound Sialin at pH 7.5 with structural model shown as cartoons and colored as in Fig 3, and map density in gray. **e** Interaction details of Fmoc-Leu-OH (as in Fig. **4c**) with the cryo-EM map densities of important residues shown at threshold 0.403.

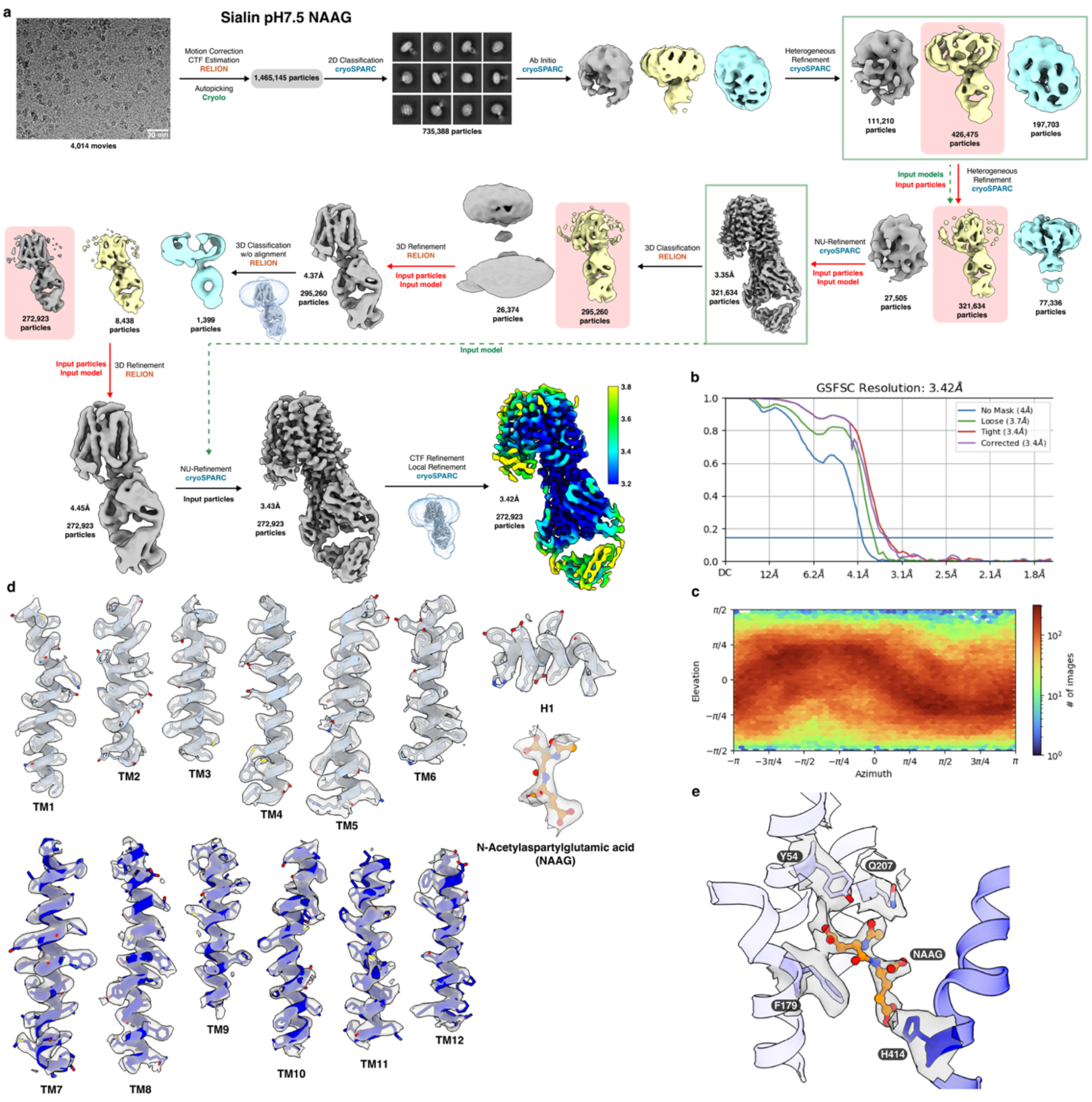

Supplementary Fig. 8: Cryo-EM workflow and analysis of NAAG-bound Sialin.

a Summary of the image processing procedures of NAAG-bound Sialin purified at pH 7.5. The final map is colored by local resolution estimation using cryoSPARC. The micrograph is a cropped representative image of the protein sample on the grid. **b** Fourier shell correlation (FSC) curves of the final reconstruction from cryoSPARC. **c** Representation of the angular distribution of the particles used in the final reconstruction from cryoSPARC. **d** Major structural features of NAAG-bound Sialin at pH 7.5 with structural model shown as cartoons and colored as in Fig 2, and map density in gray. **e** Interaction details of NAAG (as in Fig. 5c) with the cryo-EM map densities of important residues shown at threshold 0.291.

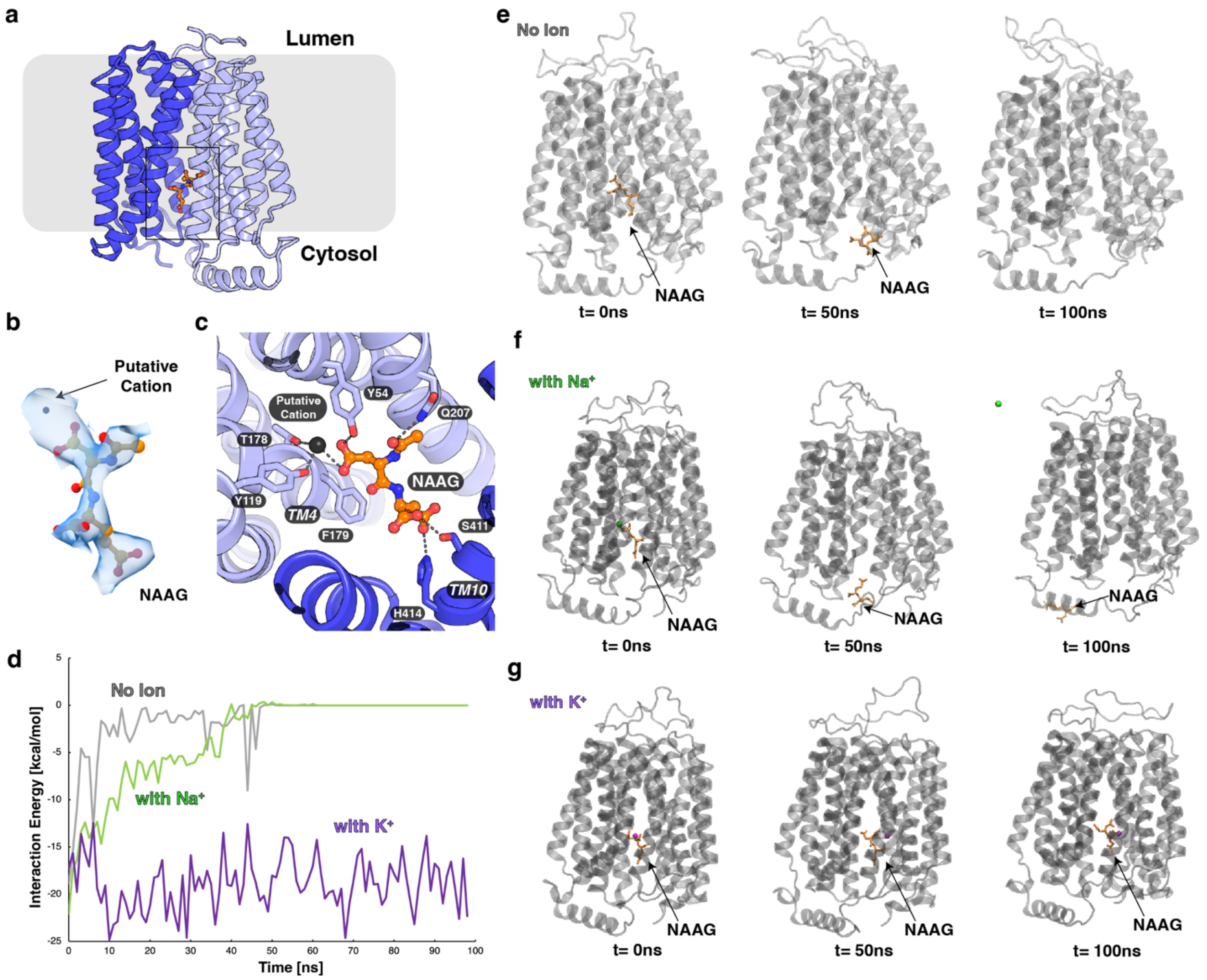

Supplementary Fig. 9: A putative cation in the NAAG binding site.

a Overall structure of NAAG-bound Sialin^{WT} viewed from the side of the membrane. **b** cryo-EM map of NAAG with the putative cation. **c** The details of the putative interactions between NAAG, cation and residues in the cavity. **d** Interaction energy between NAAG and nearby residues of Sialin without ion (gray), with sodium (green) or with potassium (purple). **e-g** Snapshots of molecular dynamics simulations at 0 ns, 50 ns, and 100 ns of the NAAG bound state without ion (**e**, Movie S2), with sodium (**f**, Movie S3) or with potassium (**g**, Movie S4) in the binding site. Without cation or sodium, NAAG fully dissociates from the binding site. Sodium is shown as a green ball and potassium is shown as a purple ball. NAAG is shown as orange sticks.

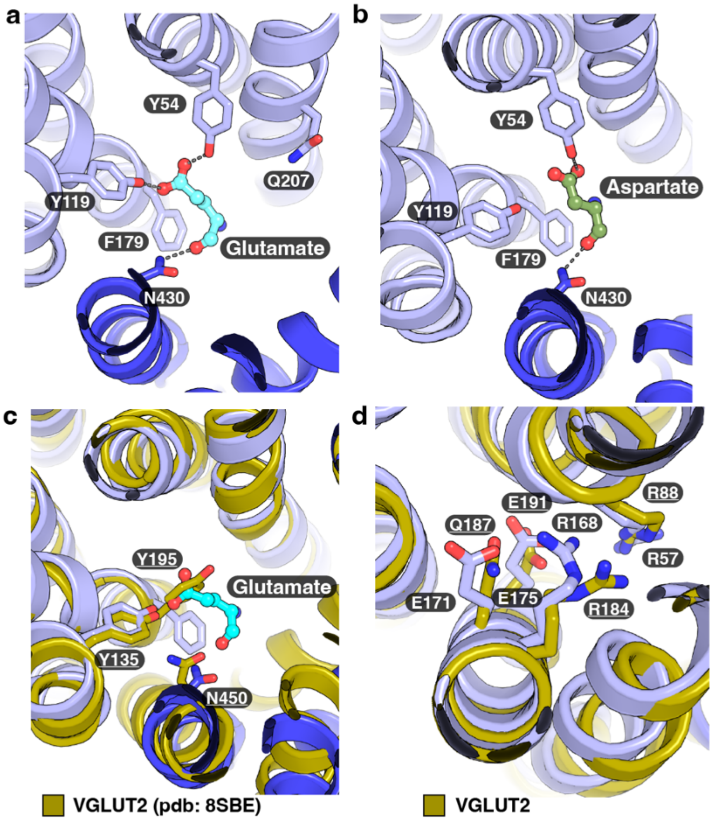

Supplementary Fig. 10: Structure models of glutamate-bound, and aspartate-bound Sialin. **a** Structure model of Sialin in complex with glutamate. **b** Structure model of Sialin in complex with aspartate. **c** Structural comparison of the modeled glutamate-bound Sialin^{WT} with apo-VGLUT2 (residues of VGLUT2 are underlined). **d** Structural comparison of the putative proton coupling residues between Sialin^{WT} and apo-VGLUT2. The related residues are labeled (residues of VGLUT2 are underlined). The hydrophilic interactions are indicated by dashed lines.

Supplementary Table 1: Cryo-EM data collection, refinement and validation statistics

	Apo Sialin pH7.5 (EMDB-41858) (PDB-8U3D)	Apo Sialin pH5.0 (EMDB-41859) (PDB-8U3E)	Apo Sialin^{R168K} (EMDB-41860) (PDB-8U3F)	NAAG-bound Sialin (EMDB-41861) (PDB-8U3G)	Fmoc-Leu-OH-bound Sialin (EMDB-41862) (PDB-8U3H)	Apo Sialin^{S61A} (EMDB-43984) (PDB-9AYB)
**Data collection and processing**						
Microscope	FEI Krios G2	FEI Krios G2	FEI Krios G2	FEI Krios G2	FEI Krios G2	FEI Krios G2
Normal Magnification	105kx	105kx	105kx	105kx	105kx	165kx
Voltage (kV)	300	300	300	300	300	300
Detector	Gatan K3	Gatan K3	Gatan K3	Gatan K3	Gatan K3	Falcon 4i
Electron exposure (e–/Å^2)	60	60	60	60	60	60
Dose rate (e-/px/s)	8.2668	8.2668	8.2668	8.2668	8.2668	8.17
Defocus range (μm)	-0.8 to -2.2	-0.8 to -2.2	-0.8 to -2.2	--0.8 to -2.2	-0.8 to -2.2	-0.8 to -2.2
Pixel size (Å)	0.83	0.83	0.83	0.83	0.83	0.738
Symmetry imposed	C1	C1	C1	C1	C1	C1
Initial particle images (no.)	379,220	288,042	721,087	1,465,145	1,078,718	441,843
Final particle images (no.)	200,101	135,283	263,841	272,923	408,146	199,874
Initial movies (no.)	1,593	1,242	3,206	4,014	4,356	2,925
Map resolution (Å)<br>FSC threshold	2.83<br>0.143	3.19<br>0.143	3.31<br>0.143	3.42<br>0.143	3.67<br>0.143	3.19<br>0.143
**Refinement**						
Initial model used (PDB code)	AlphaFold	Apo Sialin pH7.5	Apo Sialin pH7.5	Apo Sialin pH7.5	Apo Sialin pH7.5	Apo Sialin pH7.5
Model resolution (Å)<br>FSC threshold	3.04<br>0.5	3.42<br>0.5	3.47<br>0.5	3.71<br>0.5	4.01<br>0.5	3.4<br>0.5
Map sharpening *B* factor (Å^2)	-118.5	-134.4	-137.1	-172.0	-188.6	-115.1
Model composition<br>Non-hydrogen atoms<br>Protein residues<br>Ligands	 3338<br>427<br>0	 3338<br>427<br>0	 3410<br>437<br>0	 3359<br>427<br>1	 3347<br>425<br>1	 3347<br>428<br>0
B factors (Å^2)<br>Protein<br>Ligand	 52.61<br>—	 47.50<br>—	 51.22<br>—	 70.01<br>68.09	 68.77<br>76.44	 88.31<br>—
R.m.s. deviations<br>Bond lengths (Å)<br>Bond angles (°)	 0.009<br>0.774	 0.009<br>0.680	 0.005<br>0.625	 0.005<br>0.719	 0.004<br>0.778	 0.004<br>0.972
Validation<br>MolProbity score<br>Clashscore<br>Poor rotamers (%)	 1.63<br>5.37<br>0	 1.72<br>5.52<br>0	 1.38<br>6.58<br>0	 1.88<br>8.03<br>0	 1.59<br>6.12<br>0	 0.98<br>2.08<br>0.57
Ramachandran plot<br>Favored (%)<br>Allowed (%)<br>Disallowed (%)	 95.04<br>4.96<br>0	 93.62<br>6.38<br>0	 97.91<br>2.09<br>0	 93.14<br>6.86<br>0	 96.20<br>3.80<br>0	 99.06<br>0.94<br>0

Supplementary Table 2: Oligonucleotides used for cloning

OLIGO NAME	SEQUENCE	SOURCE
pFBDM_Sialin_Forward	AATGAATTCATGGACTACAAAGACGATGACGACAAGAGGTCTCCGGTTCGAGAC	Integrated DNA Technologies
pFBDM_Sialin_Reverse	ATTGCGGCCGCTCAGTGTCTGTGTCCATGGTG	Integrated DNA Technologies
Y54A_mutant_forward	TTTTTGGTTTCTTCATTGTGGCTGCATTACGTGTGAATCTGAG	Integrated DNA Technologies
Y54A_mutant_Reverse	CTCAGATTCACACGTAATGCAGCCACAATGAAGAAACCAAAAA	Integrated DNA Technologies
R57K_mutant_forward	CATTGTGTATGCATTAAAAGTGAATCTGAGTGTTG	Integrated DNA Technologies
R57K_mutant_Reverse	CAACACTCAGATTCACTTTTAATGCATACACAATG	Integrated DNA Technologies
S61A_mutant_forward	GCATTACGTGTGAATCTGGCTGTTGCGTTAGTGGATATG	Integrated DNA Technologies
S61A_mutant_Reverse	CATATCCACTAACGCAACAGCCAGATTCACACGTAATGC	Integrated DNA Technologies
Y119A_mutant_forward	TCGGTTCCTTTTTTTATGGCGCCATCATCACACAGATTCCTGG	Integrated DNA Technologies
Y119A_mutant_Reverse	CCAGGAATCTGTGTGATGATGGCGCCATAAAAAAAGGAACCGA	Integrated DNA Technologies
R168K_mutant_forward	GACCACTCATTGTACTCAAAGCACTAGAAGGACTAG	Integrated DNA Technologies
R168K_mutant_Reverse	CTAGTCCTTCTAGTGCTTTGAGTACAATGAGTGGTC	Integrated DNA Technologies
E171Q_mutant_forward	CATTGTACTCAGAGCACTACAAGGACTAGGAGAGGGTGTTAC	Integrated DNA Technologies
E171Q_mutant_Reverse	GTAACACCCTCTCCTAGTCCTTGTAGTGCTCTGAGTACAATG	Integrated DNA Technologies
E171A_mutant_forward	CATTGTACTCAGAGCACTAGCAGGACTAGGAGAGGGTGTTAC	Integrated DNA Technologies
E171A_mutant_Reverse	GTAACACCCTCTCCTAGTCCTGCTAGTGCTCTGAGTACAATG	Integrated DNA Technologies
E175Q_mutant_forward	CACTAGAAGGACTAGGACAGGGTGTTACATTTCCAG	Integrated DNA Technologies
E175Q_mutant_Reverse	CTGGAAATGTAACACCCTGTCCTAGTCCTTCTAGTG	Integrated DNA Technologies
E175A_mutant_forward	CACTAGAAGGACTAGGAGCGGGTGTTACATTTCCAG	Integrated DNA Technologies
E175A_mutant_Reverse	CTGGAAATGTAACACCCGCTCCTAGTCCTTCTAGTG	Integrated DNA Technologies
E171Q/E175Q_mutant_forward	CATTGTACTCAGAGCACTACAAGGACTAGGACAGGGTGTTAC	Integrated DNA Technologies

E171Q/E175Q_mutant_Reverse	GTAACACCCTGTCCTAGTCCTTGTAGTGCTCTGAGTACAATG	Integrated DNA Technologies
E171A/E175A_mutant_forward	CATTGTACTCAGAGCACTAGCAGGACTAGGAGCGGGTGTTAC	Integrated DNA Technologies
E171A/E175A_mutant_Reverse	GTAACACCCGCTCCTAGTCCTGCTAGTGCTCTGAGTACAATG	Integrated DNA Technologies
Q207A_mutant_forward	CATTTCATATGCAGGAGCAGCGCTTGGGACAGTAATTTC	Integrated DNA Technologies
Q207A_mutant_Reverse	GAAATTACTGTCCCAAGCGCTGCTCCTGCATATGAAATG	Integrated DNA Technologies
H298A_mutant_forward	GGGCTATCGTAGTTGCAGCCTTTTCTTACAACTGGAC	Integrated DNA Technologies
H298A_mutant_Reverse	GTCCAGTTGTAAGAAAAGGCTGCAACTACGATAGCCC	Integrated DNA Technologies
Y301A_mutant_forward	GTAGTTGCACACTTTTCTGCCAACTGGACTTTTTATAC	Integrated DNA Technologies
Y301A_mutant_Reverse	GTATAAAAAGTCCAGTTGGCAGAAAAGTGTGCAACTAC	Integrated DNA Technologies
Y306A_mutant_forward	CTTTTCTTACAACTGGACTTTTGCTACTTTATTGACATTATTGCC	Integrated DNA Technologies
Y306A_mutant_Reverse	GGCAATAATGTCAATAAAGTAGCAAAAGTCCAGTTGTAAGAAAAG	Integrated DNA Technologies
L309A_mutant_forward	CTGGACTTTTTATACTTTAGCGACATTATTGCCTACTTATATG	Integrated DNA Technologies
L309A_mutant_Reverse	CATATAAGTAGGCAATAATGTCGCTAAAGTATAAAAAGTCCAG	Integrated DNA Technologies
Y335A_mutant_forward	GTTTTTATCTTCATTGCCTGCTTTAGGCTCTTGGTTATGTATG	Integrated DNA Technologies
Y335A_mutant_Reverse	CATACATAACCAAGAGCCTAAAGCAGGCAATGAAGATAAAAAC	Integrated DNA Technologies
S411A_mutant_forward	CTTTTGCTCTTCTGGATTTGCCATCAACCATCTGGATATTG	Integrated DNA Technologies
S411A_mutant_Reverse	CAATATCCAGATGGTTGATGGCAAATCCAGAAGAGCAAAAG	Integrated DNA Technologies
H414A_mutant_forward	CTTCTGGATTTAGCATCAACGCTCTGGATATTGCTCCTTCG	Integrated DNA Technologies
H414A_mutant_Reverse	CGAAGGAGCAATATCCAGAGCGTTGATGCTAAATCCAGAAG	Integrated DNA Technologies

Supplementary Table 3: The parameters of the molecular dynamics simulations

Model	Box dimensions	Total number of atoms	Total number of water molecules	Salt concentration	Lipid composition
(1) Sialin-NAAG without a cation near NAAG	75.3 Å x 75.3 Å x 123.3 Å	64966	13891	0.15 M	100% 1-Palmitoyl-2-oleoyl-D-glycero-1-phosphatidylcholine (POPC)
(2) Sialin-NAAG with Na+ near NAAG	75.3 Å x 75.3 Å x 123.3 Å	65055	13920	0.15 M	100% 1-Palmitoyl-2-oleoyl-D-glycero-1-phosphatidylcholine (POPC)
(3) Sialin-NAAG with K+ near NAAG	75.3 Å x 75.3 Å x 123.3 Å	65055	13920	0.15 M	100% 1-Palmitoyl-2-oleoyl-D-glycero-1-phosphatidylcholine (POPC)
(4) Sialin-NANA	100.2 Å x 100.2 Å x 121.4 Å	113697	24436	0.15 M	100% 1-Palmitoyl-2-oleoyl-D-glycero-1-phosphatidylcholine (POPC)
