# Supplementary material for: Structure and inhibition of the human lysosomal transporter Sialin
Source: Nat Commun. 2024 May 23;15:4386. doi: 10.1038/s41467-024-48535-3 (PMC11116495; doi:10.1038/s41467-024-48535-3)
Supplement: Supplementary file 3 — Description of Additional Supplementary Files [file 41467_2024_48535_MOESM3_ESM.pdf]

Description of Additional Supplementary Files

File Name: Supplementary Movie 1

Description: MD simulations of NANA-bound Sialin. Simulation is shown at 100 ns time scale. Sialin is shown in gray and NANA is shown in purple sticks.

File Name: Supplementary Movie 2

Description: MD simulations of NAAG-bound Sialin with no cation. Simulation is shown at 100 ns time scale. Sialin is shown in gray and NAAG is shown in cyan sticks.

File Name: Supplementary Movie 3

Description: MD simulations of NAAG-bound Sialin with a sodium ion. Simulation is shown at 100 ns time scale. Sialin is shown in gray, NAAG is shown in cyan sticks, and sodium ion is shown as a green ball.

File Name: Supplementary Movie 4

Description: MD simulations of NAAG-bound Sialin with a potassium ion. Simulation is shown at 100 ns time scale. Sialin is shown in gray, NAAG is shown in cyan sticks, and potassium ion is shown as a magenta ball.
